# Diffusion Study by IR Micro-Imaging of Molecular Uptake and Release on Mesoporous Zeolites of Structure Type CHA and LTA

**DOI:** 10.3390/ma6072662

**Published:** 2013-07-04

**Authors:** Mauricio Rincon Bonilla, Tobias Titze, Franz Schmidt, Dirk Mehlhorn, Christian Chmelik, Rustem Valiullin, Suresh K. Bhatia, Stefan Kaskel, Ryong Ryoo, Jörg Kärger

**Affiliations:** 1 Faculty of Physics and Earth Science, University of Leipzig, Linnéstr. 5, D-04103 Leipzig, Germany; E-Mails: m.rinconbonilla@uq.edu.au (M.R.B.); titze@physik.uni-leipzig.de (T.T.); mehlhorn@physik.uni-leipzig.de (D.M.); chmelik@physik.uni-leipzig.de (C.C.); valiullin@physik.uni-leipzig.de (R.V.); 2 School of Chemical Engineering, University of Queensland, Brisbane QLD 4072, Australia; E-Mail: s.bhatia@eng.uq.edu.au; 3 Department of Inorganic Chemistry, Dresden University of Technology, Bergstrasse 66, 01069 Dresden, Germany; E-Mails: franz.schmidt@chemie.tu-dresden.de(F.S.); stefan.kaskel@chemie.tu-dresden.de (S.K.); 4 Center for Nanomaterials and Chemical Reactions, Institute for Basic Science (IBS), Daejeon 305-701, Korea; E-Mail: rryoo@kaist.ac.kr; 5 Department of Chemistry, Korea Advanced Institute of Science and Technology (KAIST), Daejeon 305-701, Korea

**Keywords:** mesoporous zeolites, IR micro-imaging, diffusion, surface barriers, NaCaA, SAPO-34, paraffins, olefins

## Abstract

The presence of mesopores in the interior of microporous particles may significantly improve their transport properties. Complementing previous macroscopic transient sorption experiments and pulsed field gradient NMR self-diffusion studies with such materials, the present study is dedicated to an in-depth study of molecular uptake and release on the individual particles of mesoporous zeolitic specimens, notably with samples of the narrow-pore structure types, CHA and LTA. The investigations are focused on determining the time constants and functional dependences of uptake and release. They include a systematic variation of the architecture of the mesopores and of the guest molecules under study as well as a comparison of transient uptake with blocked and un-blocked mesopores. In addition to accelerating intracrystalline mass transfer, transport enhancement by mesopores is found to be, possibly, also caused by a reduction of transport resistances on the particle surfaces.

## 1. Introduction

Technological application of nanoporous materials for matter upgrading [1], notably in mass separation [2] and heterogeneous catalysis [3], is based on the similarity of their pore sizes with the critical diameters of the molecules under consideration. This similarity in size leads to a dramatic retardation of molecular diffusion in comparison with the molecular bulk phase [4,5] so that mass transfer quite generally becomes one of the rate-limiting factors for their technological application [6,7].

Among the various strategies applied for overcoming this conflict, over the last few years the synthesis of nanoporous materials with hierarchical pore spaces has been under particular consideration by the community [8,9,10,11]. In addition to their content of microporous regions for ensuring their functionality for mass separation and conversion, these materials contain a network of mesopores purposefully introduced for accelerating molecular exchange between the micropores as the “active sites” for separation and conversion and the surrounding atmosphere. The success of such efforts has been demonstrated in transient sorption and release experiments with notably enhanced exchange rates [12,13,14,15,16], as well as in model reactions where the application of catalysts with hierarchical pore systems was shown to lead to significantly enhanced conversion rates [16,17,18].

In addition to its immediate technological relevance, mesopore-enhanced diffusion in nanoporous materials is also a topic of profound interest for fundamental physics. This is related to the fact that mesopore diffusion is highly sensitive to the pore phase state [19] whose establishment has remained, for more than a century, a hot topic of discussion [20,21,22,23]. Investigating history-dependent molecular diffusion in materials with hierarchical pore architecture has thus become an attractive new access to studying the equilibration process in complex pore systems [24,25].

Most of these in-depth studies of molecular diffusion in nanoporous materials with hierarchical pore spaces, notably with mesoporous zeolites, have been performed by the pulsed field gradient (PFG) technique of NMR [7,26,27,28,29]. This technique allows the rate of the redistribution of the guest molecules within the system under study to be observed [30,31], which is generally ensured to be in equilibrium. Owing to the very specific potentials of PFG NMR and its versatility, it is even possible to record molecular diffusivities separately in each of the two pore systems and to check not only overall diffusion enhancement by the presence of the mesopores, but also the opposite effect due to mesopore blocking [32].

By recording the rate of molecular redistribution, PFG NMR is unable to follow molecular equilibration during molecular uptake and release. However, experiments of this type may in fact be performed by simply following the evolution of the NMR signal [25]. The particular value of such studies is found in the ability to perform them in parallel with the PFG NMR diffusion studies. They may, simultaneously, provide enlightening insight into the molecular mobilities (by the PFG NMR studies) and into the rate of equilibration establishment. In Reference [22], this option has been exploited to demonstrate that the retardation in equilibration during sorption hysteresis is definitely not the consequence of any retardation in molecular mobilities.

NMR studies of the rate of molecular uptake and release do require, however, samples in the amount of at least several milligrams of host material. This makes them even less sensitive than some of the more recent, sophisticated techniques of uptake and release experiments [7,33], including the zero-length column (ZLC) [34,35], frequency response (FR) [36,37] and tapered-element oscillating microbalance (TEOM) [38,39,40] techniques. However, as a common feature, all these techniques are unable to record molecular uptake and release on a particular nanoporous crystal.

This option has recently been provided by the introduction of the techniques of micro-imaging [7,41,42]. By exploiting the potentials of interference microscopy (IFM) [43,44,45,46] and/or IR microscopy (IRM) [47,48,49], these techniques are able to record transient concentration profiles, notably the integrals over local concentrations in observation direction. As a prerequisite for exploiting the spatial resolution inherent to these techniques, namely of about 0.5 μm × 0.5 μm in IFM and 3 μm × 3 μm in IRM, the particles under study must be transparent and of a well-defined external shape. By using a single-element detector [41], IRM may as well be applied in the pure integral mode. In this way it is possible to follow molecular uptake or release on an individual particle. By abandoning the option of spatial resolution, IRM can now be applied with a time resolution down into the range of milliseconds. It is this mode of analysis which has been exploited in this study for investigating the transport properties of zeolites with hierarchical pore architecture.

## 2. Experimental Section

### 2.1. The Material under Study

We considered two series of specimens of 8-membered ring molecular sieves [7,50], namely materials of structure type CHA and LTA [51], each of them containing a purely microporous form and further species with differently textured mesopores.

#### 2.1.1. Silicoalumophosphate SAPO-34

The material of structure type CHA considered in this study was a crystalline silicoalumophosphate (SAPO-34) [52,53,54]. The synthesis of the material under study and its performance in adsorption kinetics and MTO-reaction is described in great detail in Reference [16]. By purposefully adding carbonaceous materials as secondary templates during the hydrothermal synthesis, samples with two different types of mesopores were fabricated; namely with mesopores separated from each other by the use of carbon nanoparticles and with a network of mesopores by the use of carbon nanotubes.

**Figure 1:**
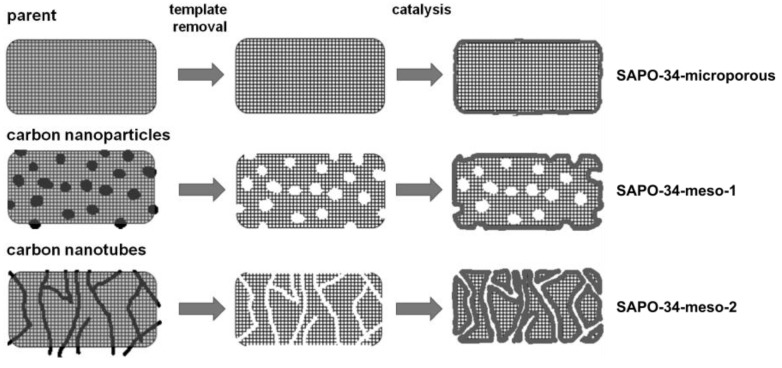
Schematics of the synthesis routes and final products of the different specimens of SAPO-34 considered in this study, adopted from Reference [16].

Figure 1 presents a schematic of the pore spaces of the three samples of SAPO-34 considered in this study and their genesis. The relevant porosity data as deduced from N_2_ physisorption and EDX measurements are summarized in Table 1. The physisorption experiments were interpreted through the multi-point BET model along with the t-plot model [16].

**Table 1:** Structure data and composition of the SAPO-34 samples under study [16].

Sample	S_BET_ (m^2^ g^−1^)	S_ext_ (m^2^ g^−1^)	V_micro_ (cm^3^ g^−1^)	V_meso_ (cm^3^ g^−1^)	Al:Si:P (mol mol^−1^)
SAPO-34-microporous	531	0	0.285	0	45:12:43
SAPO-34-meso-1	517	98	0.203	0.456	45:15:40
SAPO-34-meso-2	501	184	0.153	0.364	48:14:38

The three SAPO-34 samples under study are specified as follows:

1. The purely microporous sample (**SAPO-34-microporous**) comprises cubical-shaped crystals with a mean edge size of about 20–40 μm. However, considerable variability could be observed throughout the sample, with crystals ranging from ~10 μm–60 μm. For our tests, nearly defect-free crystals of ~30 μm edge size were chosen.
2. The carbon nanoparticle-templated sample (**SAPO-34-meso-1**) comprises cubical-shaped crystals with similar dimensions as those of SAPO-34-microporous. This sample contains spherical insertions of 20 nm diameter within the microporous framework, constituting a collection of disconnected mesopores. For our tests, crystals of ~30 μm edge size were chosen.
3. The carbon nanotube-templated sample (**SAPO-34-meso-2**) comprises irregular particles with a wide range of sizes, ranging from about 10 to 100 μm. The microporous framework is traversed by a network of mesopores forming a spanning cluster. According to the technical specification of the template carbon nanotubes, Nanocyl NC 7000, the mesopore diameters and lengths are expected to exhibit a broad distribution around mean values of about 10 nm and 1.5 μm, respectively [16].

#### 2.1.2. Zeolite LTA

**Figure 2:**
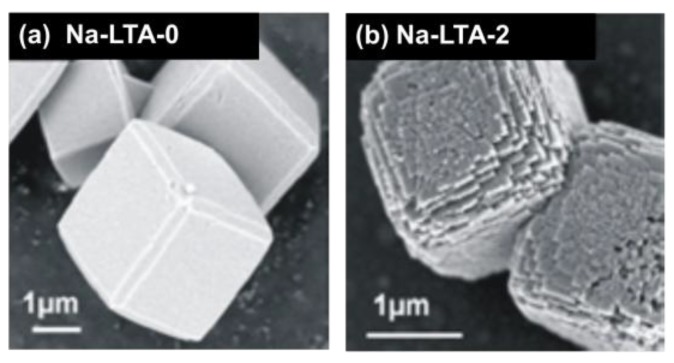
SEM images of crystals of (**a**) the purely microporous zeolite; and (**b**) the mesoporous specimen. The images were taken from the external surface of calcined samples [30].

Specimens of zeolite LTA [55,56] with mesopores included were synthesized following the procedure introduced and described in great detail in Reference [57]. The synthesis is based on the application of 3-(Trimethoxysilyl)propylhexadecyldimethylammoniumchloride (TPHAC) as an organosilane surfactant. The samples were hydrothermally synthesized at the gel compositions of 100 SiO_2_/333 Na_2_O/67.0 Al_2_O_3_/20,000 H_2_O/*n* TPHAC, with *n* = 0 for the purely microporous sample and *n* = 2 for the mesoporous one. The zeolite samples thus synthesized were denoted as Na-LTA-0 and Na-LTA-2, respectively. “Na” means the cationic form of LTA zeolite, and the numbers following “LTA” refer to the TPHAC mole numbers. From nitrogen adsorption, the total mesopore volume was estimated to be about 0.110 cm^3^/g with a mean pore width of about 5 nm. Figure 2 shows typical SEM images of the thus produced zeolite crystallites.

The signal intensity stemming from a single crystallite proved to be too small for a reliable analysis. Thus it turned out that a reasonable observation of molecular uptake and release necessitated the application of crystallite agglomerates rather than of the individual crystallites. In selected cases, for complementary uptake measurements, LTA-type crystals with sizes up to 15 μm [32] have been employed.

The Ca^2+^ ion exchange necessary for our diffusion studies with light paraffins was accomplished by dispersing 1 g of NaA-*n* in 200 mL 1 M Ca(NO_3_)_2_∙4H_2_O solution for 3 h, with magnetic stirring at 333 K. This treatment was repeated three times in all, collecting sample by filtration each time. The ion-exchanged zeolite was dried in an oven at 373 K. Finally, 82% of the sodium ions were replaced by Ca^2+^ cation exchange. Structure regularity was confirmed by X-ray diffraction as well as by field emission scanning electron microscopy (SEM, Figure 2) and transmission electron microscopy (TEM) [57]. From the micrographs, the mean crystal diameter is found to be about 2 μm. Mesopore interconnectivity was confirmed by TEM of platinum nanowires [57] which were formed within the mesopore network by adopting a procedure introduced in Reference [58]. This outcome was completely confirmed by the PFG NMR diffusion studies performed in reference [32] where the diffusion in micro- and mesopores could be shown to occur in mutually penetrating spaces.

### 2.2. IR Micro-Imaging

In IR spectroscopy, information about the amount of molecules under consideration may be deduced from the area under a characteristic IR band of the guest molecule under study [41,48,59,60,61]. In the present study, the considered host particles were neither large nor homogeneous enough for allowing the determination of spatially resolved concentration profile by use of a focal plane array detector. Instead, the IR measurements have been performed by using a single-element detector. The information thus attainable, namely the time dependence of total uptake or release, is related to that of conventional transient sorption experiments [7,35]. There is, however, a significant difference since it is now the individual host particle or crystallite rather than a bed of particles or crystallites, which is in the focus of observation. In addition to ensuring the absence of any bed resistance, uptake and release measurements with individual crystallites or particles may be also considered to be essentially unaffected by influences due to the finite rate of sorption heat release. This is the immediate consequence of the large (in fact, the largest possible) surface-to-volume ratio of the host system in such studies [62].

We have performed our experiments by use of an IR microscope (HYPERION3000, Bruker Optik GmbH, Ettlingen, Germany) which is attached to a Fourier-transform IR spectrometer (VERTEX80v, Bruker Optik GmbH, Ettlingen, Germany). The optical cell in this device is connected to a vacuum system and mounted on a movable platform under the microscope. Such an arrangement facilitates the selection of a reasonably shaped particle/crystallite for subsequent uptake and release studies. Sample activation was accomplished by heating under vacuum at a rate of 1 K/min up to 300 °C for SAPO-34 and up to 400 °C for NaCaA. The samples were kept under continued evacuation for 24 h at the maximum temperature. The subsequent uptake and release measurements have been performed after cooling down to 298 K. The transient sorption curves shown in this study are normalized, *i.e*., they represent the fractional uptake or release as a function of time, following a pressure step in the surrounding atmosphere.

Given the limited accuracy in the single-particle sorption measurements, we have restricted ourselves to comparing the measured sorption curves with the two limiting cases of diffusion-limited and barrier-limited uptake [7,35]:

(1) $$m(t)/m(\infty )=1-\frac{6}{\pi^{2}}\sum_{i=1}^{\infty }\frac{\exp(-i^{2}\pi^{2}Dt/R^{2})}{i^{2}}$$

and

(2) m(t)/m(∞)=1−exp(−3αt/R)

Here, the simplifying assumptions have been made that (i) over the considered range of guest concentrations, the intraparticle diffusivity *D* and the surface permeability *α* are constant; and that (ii) the shape of the host particles may be approached by a sphere, whose radius is determined by requiring coincidence with the volume-to-surface ratio of the crystallite/particle under study. In the purely microporous samples, *D* coincides with the intracrystalline diffusivity within the genuine micropore system.

For quantitating the rate of molecular uptake and release it is often sufficient to confine one to the first moments

(3) $$M_{1}=\int_{0}^{\infty }\left(1-m(t)/m(\infty )\right)\text{d}t$$

rather than to the complete analytical dependence as provided by Equations (1) and (2). For spherical particles one thus obtains, in the limiting cases of diffusion- and barrier-limited uptake and release [7,63],

*M*_1, diff_ = *R*^2^/15*D* (4)

and

*M*_1, bar_ = *R*/3*α* (5)

respectively. For microporous particles traversed by a network of mesopores, the intraparticle diffusivity is given by the relation [25,30,32]

*D* = *D*_micro_ + *p*_meso_*D*_meso_ (5)

here, *D*_micro(meso)_ and *p*_meso_ denote, respectively, the diffusivities in the micro-(meso-)porous spaces and the relative amount of molecules in the mesopores. For pressures far below capillary condensation as considered in our studies, the relative amount of molecules in the mesoporesis is much smaller than the amount of molecules in the micropores (*p*_meso_ << *p*_micro_ ≈ 1). Equation (6) implies the limiting case of “fast exchange” [64,65]. In this case, molecular exchange between the two pore spaces is sufficiently fast so that the molecular mean life times in either of these spaces is negligibly small in comparison with the overall observation time (*i.e.*, the time constants of molecular uptake and release as experimentally recorded).

With Equation (6), the intraparticle diffusivity is seen to be, potentially, able to unlimitedly exceed the micropore diffusivity, provided that the requirement of fast exchange between the micro- and mesopore spaces is in fact fulfilled and that the contribution *p*_meso_*D*_meso_ of mesopore diffusion to overall transport assumes correspondingly large values. The situation is completely different for mesopores separated from each other [65,66,67,68]. In this case, the presence of mesopores may only give rise to a quite moderate increase of the intraparticle diffusivity, with a factor of 2 as a typicalupper limit [25].

## 3. Results and Discussion

### 3.1. Probing Transient Sorption on SAPO-34-with-Propene

**Figure 3:**
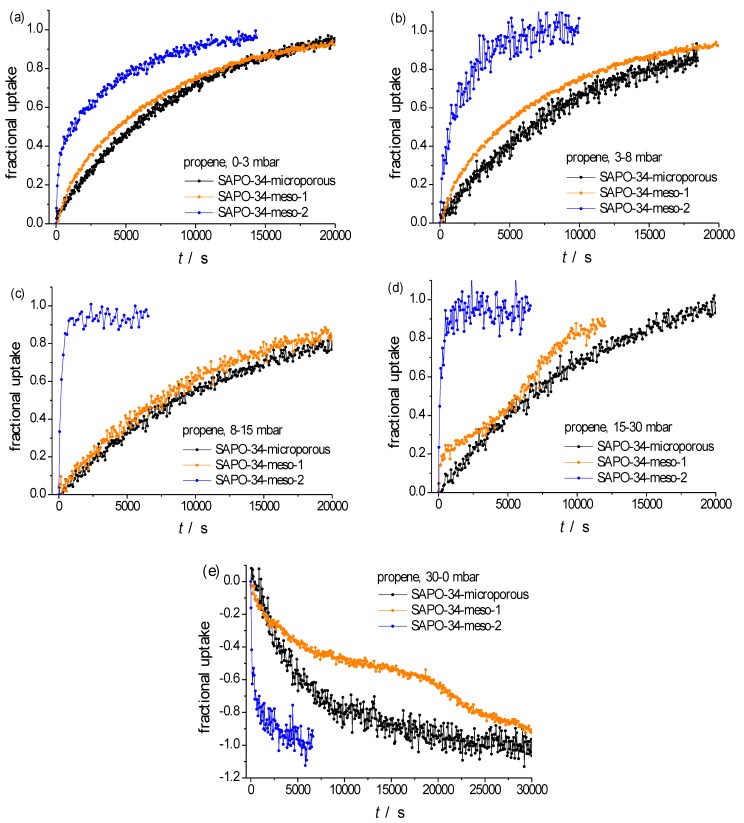
Transient sorption curves of propene at 298 K in the three different specimens of SAPO-34 considering molecular uptake initiated by pressure steps from (**a**) 0–3 mbar; (**b**) 3–8 mbar; (**c**) 8–15 mbar; (**d**) 15–30 mbar; and (**e**) release by a pressure step from 30 to 0 mbar. The particles were selected to have essentially coinciding effective radii, namely about 15 μm for SAPO-34-microporous and -meso-1 and about 17 μm for SAPO-34-meso-2.

For investigating molecular uptake and release simultaneously in all three specimens, propene offered by far the best measuring conditions. A survey about the thus attained sorption curves is provided by Figure 3. It nicely reproduces the finding reported, in a qualitative way, already in Reference [16] on introducing these very specimens: While the incorporation of mutually separated mesopores (SAPO-34-meso-1) does lead to not more than a quite moderate acceleration of molecular uptake in comparison with the purely microscopic sample, molecular uptake in SAPO-34-meso-2, accommodating a complete network of mesopores (see Figure 1), is most dramatically accelerated. This effect increases with increasing pressures (compare Figure 3a–d) and is also visible during molecular release (Figure 3e).

**Figure 4:**
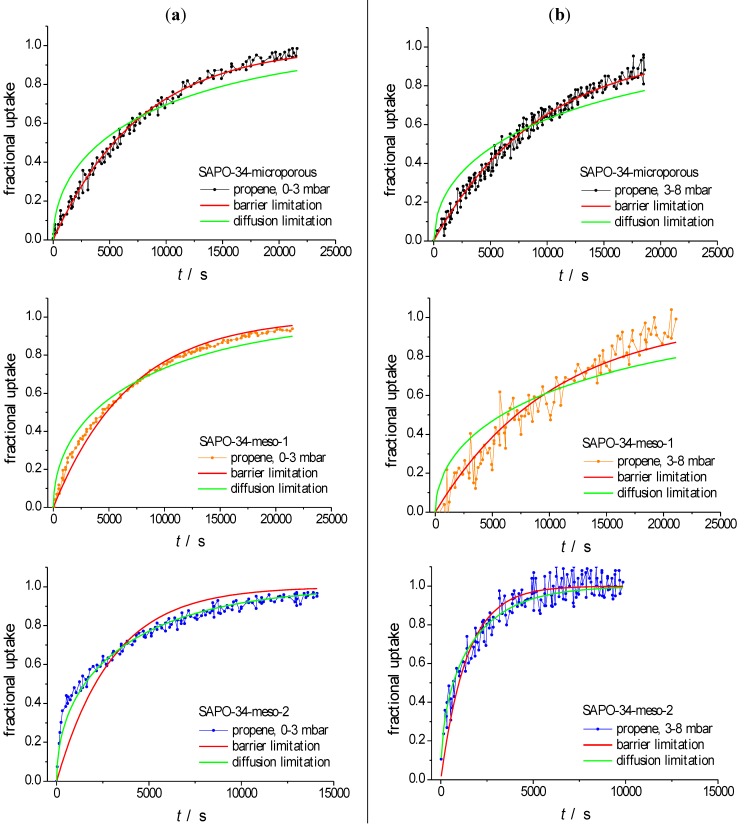

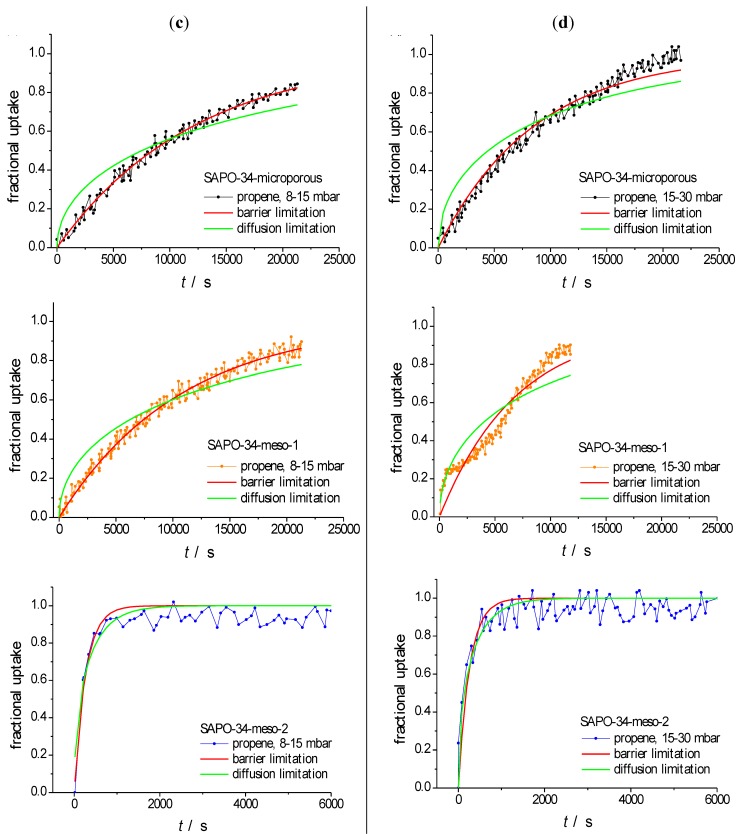

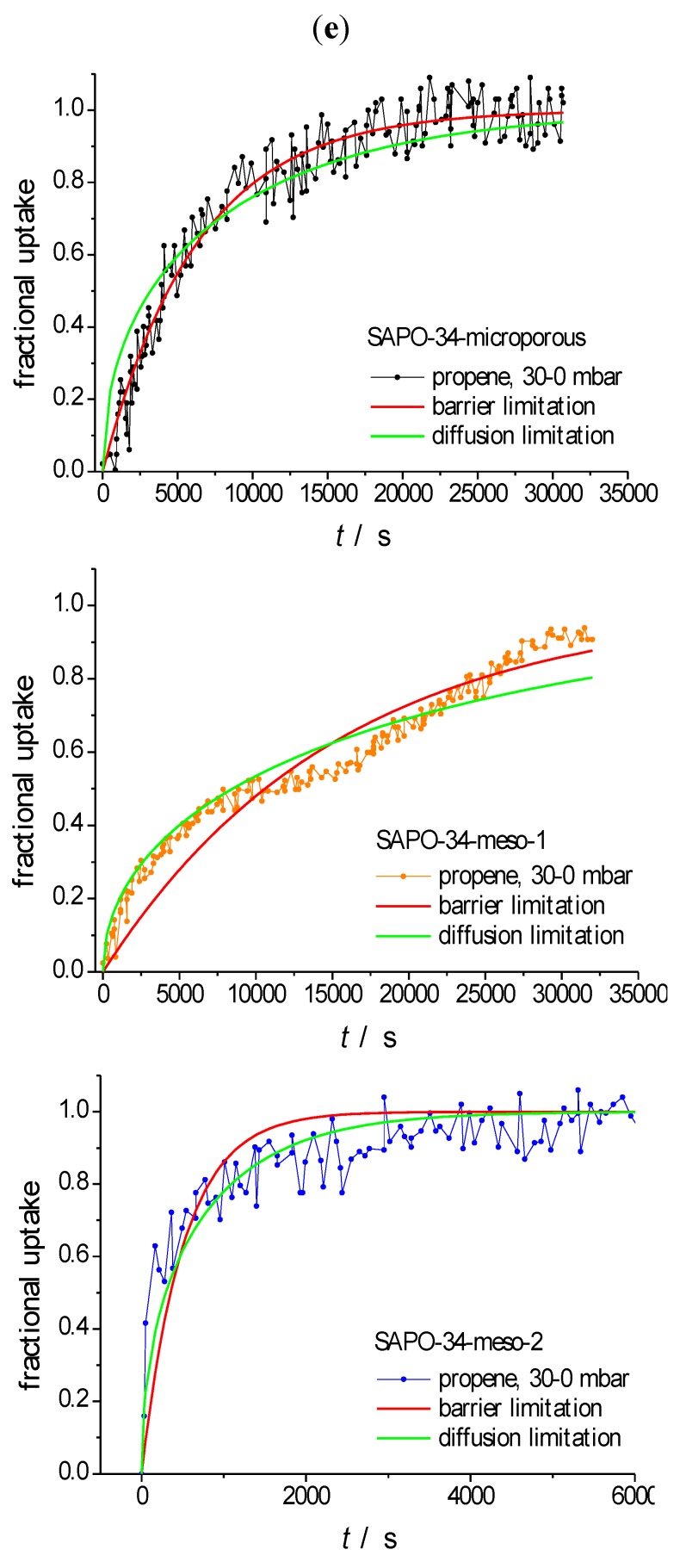
Fitting of uptake curves of propene in SAPO-34-microporous (top), SAPO-34-meso-1 (centre) and SAPO-34-meso-2 (bottom) at 298 K. The green (red) curves represent the results obtained by fitting with the theoretical dependences expected for limitation by diffusion, Equation (1), (surface permeation, Equation (2)). (**a**) 0–3 mbar; (**b**) 3–8 mbar; (**c**) 8–15 mbar; (**d**) 15–30 mbar; and (**e**) 30–0 mbar.

For facilitating the further discussion, Figure 4 provides a comparison between the experimental data of Figure 3 with the dependences theoretically expected for molecular uptake and release in the limiting cases of complete control by surface resistances and by intracrystalline diffusion.

Note the fair agreement appearing from Figure 4 for the purely microporous sample (left column) between the experimental data and the theoretical dependences for barrier-limited uptake and release (suggesting that, for this sample, the *α* values (Table 2) have the meaning of genuine surface permeabilities while the *D* values turn out to be mere fitting parameters which we refer to as apparent diffusivities). It is not unexpected, therefore, that these values are notably (namely by at least one order of magnitude) exceeded by the literature data [50,69]. Transport resistances caused by surface resistances are quite commonly observed in nanoporous host-guest systems and known to be affected by sample synthesis, storage and pretreatment in often quite complex ways [70,71,72,73,74,75,76,77,78]. Conversely, transient sorption on SAPO-34-meso-2 is seen to be best approached by assuming diffusion limitation so that in this case the *D* values (Table 2) are assumed to represent genuine intracrystalline diffusivities. From molecular uptake and release with SAPO-34-meso-1 (central column) no clear assessment is possible.

**Table 2:** Fitting parameters used for the representation of the analytical expressions shown in Figure 4 for diffusion-limited (*D* in Table 2, fit to Equation 1) and for barrier-limited (*α* in Table 2, fit to Equation 2) uptake and release of propene in various specimens of SAPO-34 at 298 K, as a function of the pressure step. Fitting parameters which, by comparison between experimental measurement and theoretical dependence, may be considered as physically meaningful quantities (*i.e.*, intraparticle diffusivities or surface permeabilities) are printed in bold.

Transport parameter	Host material	Pressure step (mbar)				
		0–3	3–8	8–15	15–30	30–0
***D*** **×** **10^14^ (m^2^** **s^−1^)**	SAPO-34-microporous	0.266	0.197	0.140	0.234	0.339
	SAPO-34-meso-1	0.301	0.177	0.181	0.264	0.120
	**SAPO-34-meso-2**	**1.120**	**2.540**	**13.000**	**13.400**	**5.400**
***α* × 10^10^ (m** **s^−1^)**	**SAPO-34-microporous**	**7.94**	**6.57**	**4.95**	**7.14**	**2.61**
	SAPO-34-meso-1	8.85	5.83	5.92	9.00	3.87
	SAPO-34-meso-2	26.10	56.80	330.00	333.00	150.00

In recent comparative studies of surface permeation and intracrystalline diffusion by micro-imaging in both LTA-type zeolites [79,80] and MOFs of type Zn(tbip) [45,46] the formation of surface barriers could be referred to the total blockage of an overwhelming part of the micropores connecting the intra-particle pore space with the surrounding, rather than to the existence of a quasi-homogeneous layer of extremely reduced permeability on the external surface.

It is noteworthy that uptake and release on SAPO-34-meso-1 occurs with similar time constants as on the purely microporous samples. This finding is not unexpected due to the following two reasons: The existence of mesopores dispersed within the particle bulk phase cannot give rise to a decisive decrease of the surface resistances (which, for the purely microporous sample, were found to be rate-limiting for molecular uptake and release). Moreover, even under the conditions of diffusion limitation, *i.e.*, for completely missing surface resistances, dispersed mesopores are known to lead, if at all, to only a quite moderate increase in the overall diffusivity [25,65,81]. It is noteworthy, however, that the time dependence of molecular uptake and release in SAPO-34-meso-1 does not consistently follow anymore the pattern of barrier limitation. There is, for uptake at larger pressures, even the indication of a two-stage behavior. We relate these peculiarities to some structure which evolved during mesopore generation in these samples.

In addition to giving rise to much faster uptake and release curves, the incorporation of a network of mesopores in SAPO-34-meso-2 (instead of a “swarm” of mesopores separated from each other as in SAPO-34-meso-1) is seen to lead to a notable change in the time dependence. It is now nicely seen to approach the pattern of diffusion-limited uptake. The presence of mesopores extending up to the particle surface does, obviously, ensure a sufficiently fast exchange between the intra- and extra-particle spaces so that micropore blockage close to the external surface cannot be expected anymore to give rise to a significant retardation of mass transfer. Not unexpectedly, the resulting diffusivities as summarized in Table 2 are seen to increase dramatically with the increase in the mean value of pressure and, hence, of loading covered in the transient sorption experiments. In fact, either of the two terms on the right hand side of Equation (6) is easily seen to increase with increasing pressure or loading: As a first-order estimate, the concentration dependence of the intracrystaline (transport or Fickian) diffusivity *D*_micro_ may be approached by that of the “thermodynamic factor” dln*p*/dln*c* [7,82] which, for Langmuir-type isotherms (as relevant for the system under consideration [50,69]), would yield

(7) $$D\left(c\right)=D_{0}\frac{\text{dln}p}{\text{dln}c}=D_{0}\frac{1}{1-c/c_{\text{max}}}$$

with *p*(*c*) denoting the gas phase pressure in equilibrium with the sorbate concentration *c* and *c*_max_ the loading at saturation; *D*_micro_, the first term in Equation (6), is thus seen to increase with pressure and/or loading. *D*_meso_ is given by the Knudsen diffusivity which, in the mesopores of typically 10 nm diameter, may be assumed to be independent of loading. The increase of the second term in Equation (6) is hence easily seen to result in consequence of the increase of the pressure in the mesopores, leading (beyond the Henry region of smallest concentrations) to an increase of the relative amount *p*_meso_ of molecules in the mesopores.

Additional insight into the mechanisms of mass transfer in the mesoporous SAPO-meso-2 may be achieved by comparing the experimentally determined diffusivities (Table 2) with an estimate of the contribution of mesopore diffusion, *p*_meso_*D*_meso_, as expected on the basis of literature data. Noting that *p*_meso_ is given by the ratio *N*_meso_/(*N*_meso_ + *N*_micro_), where *N*_meso_ and *N*_micro_ are, respectively, the numbers of molecules in meso- and micropores at given external conditions, it is straightforward to show that

(8) $$p_{meso}\approx \frac{pV_{meso}^{2}}{qV_{micro}RT}$$

where *p* is the gas pressure; *q* is the adsorption; *R* is the universal gas constant; and *T* is temperature.

For estimating the diffusivity *D*_meso_ in the mesopore space we use the Knudsen relation [83,84,85,86]

(9) $$D_{meso}=\frac{1}{3}dv$$

with *d* and v denoting, respectively, the mean pore diameter and the thermal velocity of the guest molecules; With values of *p* = 30 mbar; *V*_micro_ and *V*_meso_ as given in Table 1; and *q* ≈ 7.0 × 10^−4^ mol/g [50,69] and by implying a perfectly interconnected channel system of mesopores, *p*_meso_*D*_meso_ for propene in SAPO-meso-2 thus results to be of the order of 10^−9^ m^2^ s^−1^. This estimate dramatically exceeds the overall diffusivity (of order 10^−14^ m^2^ s^−1^ to 10^−13^ m^2^ s^−1^) determined from molecular uptake. The fast-exchange relation, Equation (6), is thus seen to fail in adequately reflecting transport enhancement in mesoporous SAPO-meso-2.

For the further discussion, we consider the opposite limiting case and assume that molecular uptake occurs in a sequence of two processes, namely fast equilibration between the mesopores and the surrounding atmosphere in response to the pressure change in the surrounding atmosphere, followed by the much slower process of equilibration between the mesopores and the microporous bulk phase. Knudsen diffusion and micropore diffusion are easily seen to be the governing mechanisms in these processes and it is the time constant of the second process which controls the rate of the overall process. On the basis of these model considerations, the data of Table 2 for propene diffusion in SAPO-34-meso-2 do, obviously, require a modified interpretation. By abandoning the fast-exchange model, the SAPO-34-meso-2 particles cannot be considered to represent a quasi-homogeneous continuum with a well-defined radius *R*. Instead of giving rise to a second term in the effective diffusivity (representing the contribution of mass transfer in the mesopores to overall diffusion) transport enhancement by the presence of mesopores is then rather found to be caused by a reduction of the extension of the purely microporous regions which, upon adsorption, are filled by the guest molecules (or, correspondingly, are depleted upon desorption). By inserting the respective time constants of molecular uptake and release into Equation (4) and taking *R* as a measure of the extension of these purely microporous regions, with the data of Table 2 the incorporation of mesopores may thus be estimated to lead, in comparison with the purely microporous particles, to a reduction of the extension of these purely microporous regions by about one order of magnitude. Since for barrier limitation (Equation (5)) the time constant does scale with *R* rather than *R*^2^, in this case the reduction in the extension of the purely microporous regions may be estimated to amount to even two orders of magnitude.

### 3.2. Fast Uptake and Release on SAPO-34 with Ethane and Intentional Barrier Formation

**Figure 5:**
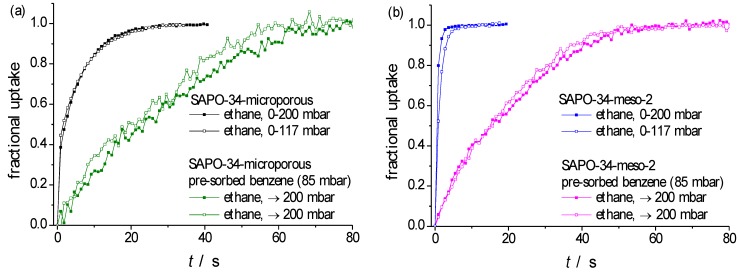
Uptake curves of ethane at 298 K in (**a**) SAPO-34-microporous; and (**b**) SAPO-34-meso-2. The black and blue curves represent the uptake curves for pure ethane using pressure steps of 0–200 mbar and 0–117 mbar, respectively. The green and magenta curves represent two runs of the following experiment: benzene is initially fed into the cell at a pressure of 85 mbar. Subsequently, a step increase in ethane pressure is produced so that the total pressure of the bulk phase increases to 200 mbar.

**Figure 6:**
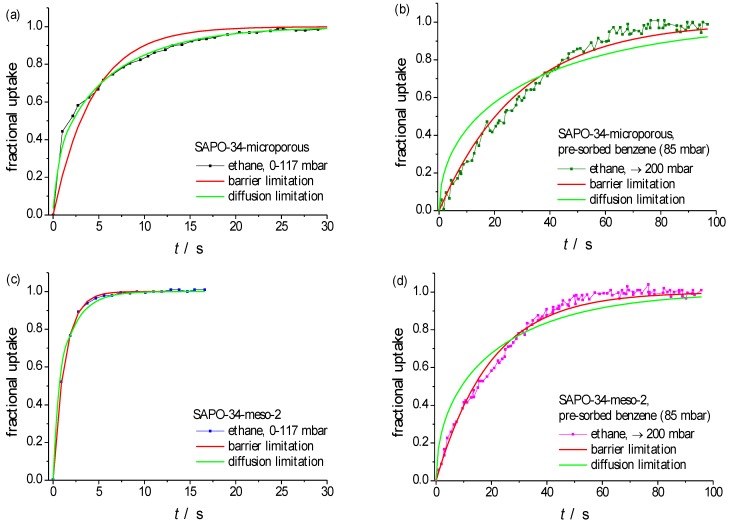
Comparison of the experimentally observed uptake curves (see Figure 5) of (**a**,**b**) ethane at 298 K in purely microporous SAPO-34; (**c**,**d**) in SAPO-34-meso-2; following an ethane pressure step from (**a**,**c**) 0 to 117 mbar; and (**b**,**d**) after benzene pre-adsorption (equilibration at 85 mbar), with subsequent pressure enhancement to 200 mbar by introducing ethane into the sorption vessel. Uptake curves obtained by fitting for diffusion limitation (Equation (1)) are shown in green and for barrier limitation (Equation (2)) in red. The fitting parameters are summarized in Table 3.

Figure 5 displays uptake curves on a particle of purely microporous SAPO-34 and of SAPO-34-meso-2, both with a mean radius *R* ≈ 25 μm, considering ethane as a probe molecule. Following the procedure applied already to the transient sorption curves with propene (Figure 3 and Figure 4, Table 2), Figure 6 provides a comparison of the recorded curves with the theoretical dependences resulting in the limiting cases of either diffusion-limited (Equation 1, green lines) or barrier-limited (Equation 2, red lines) uptake, with the fitting parameters summarized in Table 3. Again, those parameters (diffusivities or surface permeabilities) which—by yielding satisfactory fits to the experimental data—may be assumed to represent physically reasonable quantities, are printed in bold.

It is noteworthy that the strong surface barriers observed on the purely microporous specimens using propene as a guest molecule do not occur anymore for ethane. The ethane molecules are thus seen to be able to penetrate particle areas close to the surface which, obviously, are essentially impermeable for the notably larger propylene molecules.

For estimating the contribution of mesopore diffusion to overall mass transfer in the fast-exchange model, Equation (6), we have used *p* = 200 mbar and *q* ≈ 6.0 × 10^−4^ mol/g [50,69] and apply, once again, the Knudsen relation, Equation (9) as a first-order estimate, implying a well-connected system of mesopores. Similarly as observed already in Section 3.1 with propene, the resulting value of *p*_meso_*D*_meso_ ≈ 10^−8^ m^2^ s^−1^ is much larger than the diffusivities as resulting from the rate of molecular uptake and release (Table 3). Also, by using ethane as a probe molecule, fast exchange between the micro- and mesoporous spaces, *i.e.*, the simultaneous contribution of mass transfer in the micropores and mesopores to overall diffusion is thus found to be unable to provide a self-consistent explanation of transport enhancement in SAPO-meso-2. It is thus rather the reduction of the extension of the purely microporous regions brought about by the network of mesopores which one should consider as the main origin of the dramatic reduction in the time constants of molecular uptake and release in SAPO-meso-2 (see Equations (4) and (5), with *R* taken as a measure of the size of these regions) which we have observed in our experiments.

**Table 3:** Fitting parameters yielding best approach to the experimental data displayed in Figure 5 by implying diffusion-limited (*D* in Table 3, fit to Equation (1)) and barrier-limited (*α* in Table 3, fit to Equation (2)) uptake and release for ethane in SAPO-34-microporous and SAPO-34-meso-2. Fitting parameters which, by comparison between experimental measurement and theoretical dependence, may be considered as physically meaningful quantities (*i.e*., intraparticle diffusivities or surface permeabilities, see the examples given in Figure 6) are printed in bold.

Transport parameter	Host material	Pressure step (mbar)			
		0–117	0–200	0–200; pre-sat. with Benzene 85 mbar Run 1	0–200; pre-sat. with Benzene 85 mbar Run 2
***D*** **×** **10^12^ (m^2^ s^−1^)**	**SAPO-34-microporous**	**4.95**	**4.85**	0.756	0.918
	**SAPO-34-meso-2**	**83.8**	**96.6**	5.320	5.55
***α*** ****** **×** **10^7^ (m s^−1^)**	**SAPO-34-microporous**	5.67	73.2	**2.13**	**2.52**
	**SAPO-34-meso-2**	104.84	125.5	**66.9**	**70.6**

In the present studies, in addition to recording molecular uptake by the freshly activated sample, a second set of experiments has been performed. These experiments were started by equilibrating the sample with a second component (benzene) whose critical molecular diameter (~0.6 nm) is large enough to exclude any adsorption in the micropores (with pore openings of 0.38 nm × 0.43 nm). Moreover, by using benzene as a second component any disturbing interference with the IR signal of the probe molecules under study (ethane) could be avoided. The benzene pressure in the surrounding atmosphere (84 mbar) was chosen to be large enough for ensuring mesopore saturation. Subsequently, the atmosphere in the sorption vessel was complemented with ethane, up to the establishment of a total pressure of 200 mbar. The subsequently recorded uptake curves are as well included in Figure 5 and Figure 6. In addition to a dramatic retardation in the uptake rates, benzene pre-adsorption on the purely microporous sample is found to give rise to a clear shift in the shape of the uptake curve from diffusion limitation (Figure 6a) to barrier limitation (Figure 6b).

The appearance of a surface barrier may be easily referred to the formation of a layer of liquid benzene on the external surface of the particle which is formed as a simple consequence of the enhancement in benzene pressure above saturation pressure (~140 mbar) close to the particle surface, given the fact that the overall pressure enhancement to 200 mbar is not accompanied by a sufficiently rapid mixing of the gas phase within the sorption vessel. This reasoning is in nice agreement with the observation that, after benzene pre-sorption, molecular uptake on the purely microporous sample occurs with a similar rate as on SAPO-34-meso-2.

For an order-of-magnitude estimate of the effect of a liquid film on surface-permeation we quote the standard Equation [7,81]

*α* = *p*_f_*D*_f_/*d* (10)

for the permeability through a surface film of thickness *d*_f_; with the (host-related) solubility *p*_f_; and the diffusivity *D*_f_ of the guest molecules in the liquid film. For a rough estimate, we approach the ethane diffusivity in liquid benzene with the benzene diffusivity (*D* ≈ 2 × 10^−9^ m^2^ s^−1^ [87]). In Reference [88], the ethane solubility in liquid benzene (in mole fractions, related to the ethane gas phase at a pressure of 1 atm) has been determined as ≈ 0.014. With an ethane guest concentration of 2 mmol/g in SAPO-34 at 1 atm [50], this leads to *p*_f_ ≈ 10^−4^. With Equation (10), a value of *α* = 2.13 × 10^−7^ m s^−1^ as resulting in our measurements for the permeability through the liquid films (Table 3) is found to correspond to a film thickness of about 1 μm which turns out to be of a quite reasonable order of magnitude.

### 3.3. Uptake and Release on Different LTA Specimens Using Ethane and Propane as Probe Molecules

Complementing the series of IRM measurements of molecular uptake and release on individual particles of various specimens of SAPO-34, we have applied the same technique for investigating different (namely purely microporous and mesoporous) specimens of zeolite LTA. We have considered these very samples, which were in the focus of first in-depth studies of diffusion in mesoporous zeolites, by using the pulsed field gradient (PFG) technique of NMR. The samples under consideration consisted of both small crystallites (as exemplified in Figure 2, see Reference [30]) and larger ones with mean diameters up to the order of 15 μm [31,32].

**Figure 7:**
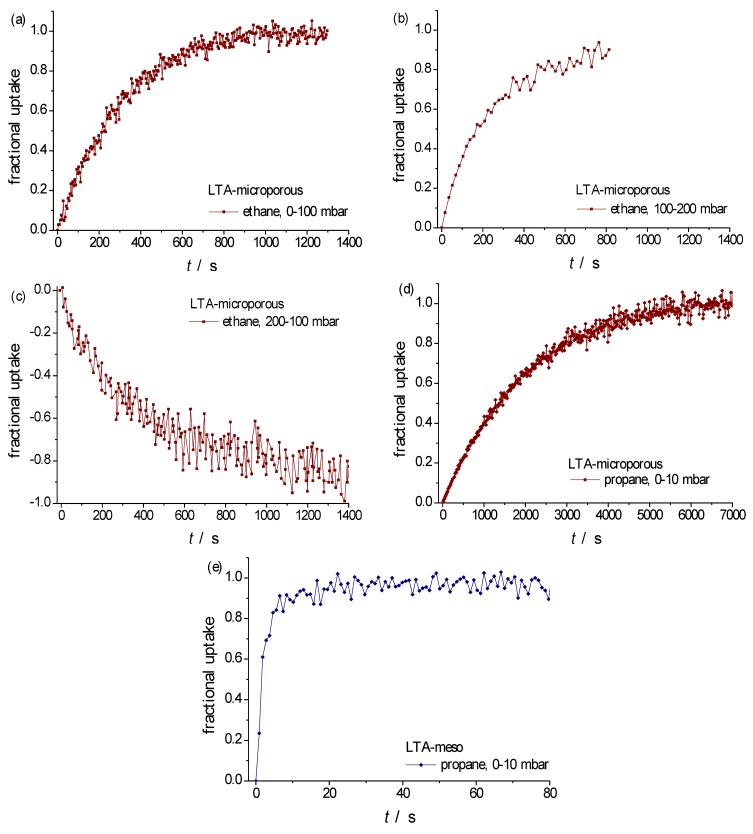
(**a**,**b**,**d**) Molecular uptake; (**c**) and release with ethane and propane at 298 K in the specimens of purely microporous zeolite LTA (NaCaA-LTA-0) under the conditions given in the insets; (**e**) and with propane in mesoporous LTA (NaCaA-LTA-2). For both samples, the selected particles were agglomerates with a radius of the order of 15μm, consisting of crystallites with diameters in the μm range.

Figure 7 provides examples of molecular uptake and release observed with agglomerates of LTA-type crystallites both in the purely microporous form (Figure 7a–d) and in a mesoporous species (Figure 7e). The agglomerate radii were of the order of *R* ≈ 15 μm, the sizes of the individual crystallites in the μm range. With literature data for the diffusivities of ethane (~10^−10^ m^2^ s^−1^ [7,89]) and propane (~10^−13^ m^2^ s^−1^ [7,89]) in purely microporous LTA of comparable cation content, by means of Equation (4) the time constants for diffusion-limited uptake and release on the individual crystallites can be estimated to be of the order of milliseconds and seconds, respectively. By contrast, the experimentally determined uptake and release times are found to be in the range of hundreds of seconds for ethane (Figure 7a–c) and of thousands of seconds for propane (Figure 7d), providing clear evidence of barrier-limited uptake and release.

The dramatic transport enhancement documented in Figure 7e for propane in mesoporousLTA—appearing in sorption time constants of seconds rather than thousands of seconds—is thus seen to be also brought about by the elimination of surface resistances, in addition to the creation of “highways” of mass transfer due to the incorporation of mesopores. It is noteworthy that the PFG NMR diffusion studies with the same material reported in Reference [30] did also refer to the option of barrier-limited mass transfer in the purely microporous specimens of LTA. In Reference [32], PFG NMR studies with particles with notably larger diameters (up to 15 μm) allowed the unambiguous determination of intracrystalline diffusivities. In these studies, transport enhancement in mesoporous zeolites could be attributed to the sole influence of the mesopores (following the fast-exchange relation, Equation (6)) so that also the purely microporous species in this series were assumed to be free of any significant surface resistance.

**Figure 8:**
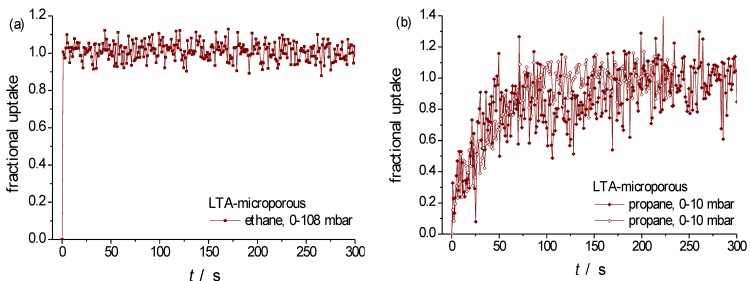

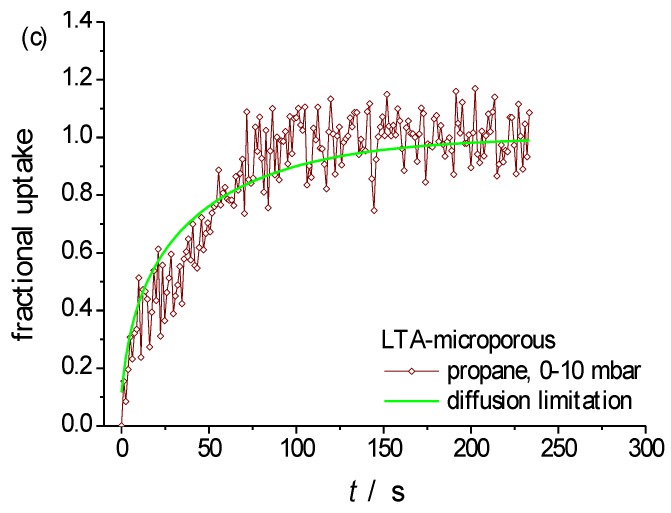
Uptake of (**a**) ethane; (**b**) propane (two different symbols, corresponding to two different runs) at 298 K on a single crystal of type NaCaA-0; and (**c**) fitting of propane uptake (one run) by implying diffusion limitation (Equation (1)).

Figure 8 shows the transient uptake curves, determined with a single crystal of purely microporous zeolite LTA for ethane (Figure 8a) and propane (Figure 8b), with a mean radius of *R* Ɉ 8 μm. For ethane, molecular uptake is found to occur with a time constant of smaller than 1 s. With Equation (4), the intracrystalline diffusivity may thus be estimated to be larger than 10^−11^ m^2^ s^−1^ which is in complete agreement with the above quoted literature data. Figure 8c compares the experimentally determined uptake data with the theoretical dependence expected for diffusion-limited uptake (Equation (1)) where the best fit has been obtained by assuming an intracrystalline diffusivity of *D* = 1.4 × 10^−13^ m^2^ s^−1^. This value is seen to be in excellent agreement with the literature data, providing clear evidence that molecular uptake and release by the larger crystallites is dominated by intracrystalline diffusion.

## 4. Summary and Conclusions

Deviating from the conventional techniques of transient sorption measurements which are confined to the investigation of compacted nanoporous material or beds of particles, micro-imaging by IR microscopy allows monitoring molecular uptake and release on individual particles. In this way, disturbing influences by external transport resistances and a finite rate of sorption heat release can be circumvented. Moreover, being sensitive to a certain band in the IR spectra of the molecules under study, IR micro-imaging is as well applicable to selective diffusion measurements in multi-component systems. This includes the application of molecules with different sizes so that the larger one can only be accommodated by the larger pores. In this way, mass transfer of the smaller molecules in the larger pores can be deliberately affected by the presence of the larger molecules, while mass transfer in the smaller pores remains unaffected.

The present study was dedicated to the exploitation of differences in molecular uptake and release in narrow-pore host species (SAPO-34 and NaCa-LTA) on comparing the purely microporous species with their mesoporous counterparts, *i.e.*, host materials containing a system of mesopores in addition to the micropore space. The guest molecules (ethane, propene and propane) were selected to ensure particularly suitable experimental conditions for discriminating the differences in the transport properties of the samples under study.

In complete agreement with our expectation based on simulations of mass transfer in such systems [25,65] and with previous measurements of both this very material SAPO-34-meso-1 [16] and other host systems of comparable pore structure [67,68], there was not more than a modest acceleration by the presence of mesopores separated from each other. Networks of mesopores incorporated in the microporous bulk phase, however, gave rise to a remarkable increase in molecular uptake and release. It was interesting to find that—in addition to the creation of new diffusion paths (“highways of mass transfer”) in the interior of the particles—transport enhancement by the incorporation of a network of mesopores could have been also a consequence of notably enhanced surface permeabilities. Uptake and release studies with nanoporous materials with varying surface permeabilities might thus prove to become a new tool for the exploration of surface resistances. Given the fact that our knowledge about their origin of surface barriers, the mechanisms of variation and their impact on mass transfer is still rather limited [45,46,70,71,72,73,74,75,76,77,78,79,80,90,91,92,93] any new type of information which, in this way, may become attainable is more than welcome.

For SAPO-34, these enhancements in surface permeability (corresponding with vanishing surface resistances) could be shown to nicely appear in a pronounced shift of the shape of the transient sorption curves from barrier limitation to diffusion limitation. Exactly the reverse behavior was observed in uptake experiments with ethane, following pre-saturation experiments with benzene. With these types of experiments we adopted, in some way, the procedure employed in Reference [32] for exploring the micropore diffusion in mesoporous zeolites with blocked mesopores. While in Reference [32], by exploiting the potentials of PFG NMR, the distinction between the two species was ensured by using proton-containing and deuterated guest molecules, in the present study this distinction was based on differences in the IR spectra of benzene and ethane. Ethane uptake following benzene pre-sorption did turn out, however, to be limited by the formation of a liquid film on the external particle surface, corrupting any information about intracrystalline mass transfer. As an immediate consequence of this change in the dominating transport resistances, molecular uptake on the purely microporous and mesoporous particles (implying similar sizes) is found to proceed at essentially identical rates. In addition, the shape of the transient sorption curves is again seen to follow the pattern of barrier limitation. This agreement between the shape of the uptake curve and the dependence expected on the basis of the governing mechanism nicely confirms the validity of our conclusion to take a similar shape of the uptake curves in purely microporous SAPO-34 (Figure 4 left column) as an indication for the dominance of surface barriers.

Deviating from the observation with the specimens of mesoporous zeolites, NaCaA (Section 3.3 and Reference [31,32]), transport enhancement in SAPO-34 with incorporated mesopore networks(SAPO-34-meso-2) was less pronounced than predicted by applying the fast-exchange model, *i.e.*, by assuming that molecular uptake and release are accomplished by “parallel” fluxes in the micro- and mesopores. In fact, the estimate of the contribution of mass transfer in the mesopores (*p*_meso_*D*_meso_) to overall diffusion (Equation (6)) leads to values which exceeded the diffusivities as resulting from molecular uptake and release on the individual particles by orders of magnitude. Enhancement in the uptake and release rates thus appears to be rather a consequence of the reduction of the extension of the purely microporous regions brought about by the network of mesopores.

One has to have in mind, however, that the estimate of *p*_meso_*D*_meso_ has been based on the supposition of an ideal, well-connected channel system of mesopores. Given the finite length of about 1.5 μm of the templating carbon nanotubes, however, it is not unlikely that there are also constrictions or even interruptions in the mesopore channel system so that, by considering these influences, an estimate of *p*_meso_*D*_meso_ would lead to notably smaller values, possibly leading to an even complete agreement with the experimental results of transport enhancement.

A final decision about the prevailing mechanism leading to the difference between the experimentally observed and the theoretically estimated degree of transport enhancement by the presence of mesopores in SAPO-34-meso-2 cannot be given on the basis of the available data. In either case, however, there is clear evidence of potentials for further transport enhancement by the incorporation of further advanced mesopore systems [94,95,96,97]. Combination of experimental measurement with the message of micro-kinetic estimates is thus, once again, seen to serve as an important source of technological development.
